# A case of convergent-gene interference in the budding yeast knockout library causing chromosome instability

**DOI:** 10.1093/g3journal/jkab084

**Published:** 2021-03-16

**Authors:** Molly R Gordon, Jin Zhu, Victoria Qu, Rong Li

**Affiliations:** 1 Department of Cell Biology, Center for Cell Dynamics, Johns Hopkins University School of Medicine, Baltimore, MD 21205, USA; 2 Department of Chemical and Biomolecular Engineering, Whiting School of Engineering, Johns Hopkins University, Baltimore, MD 21218, USA; 3 Mechanobiology Institute and Department of Biological Sciences, National University of Singapore, Singapore 117411, Singapore

**Keywords:** *SNO1*, *CTF13*, chromosome instability, YKO collection, convergent gene pairs

## Abstract

To maintain genome stability, organisms depend on faithful chromosome segregation, a process affected by diverse genetic pathways, some of which are not directly linked to mitosis. In this study, we set out to explore one such pathway represented by an undercharacterized gene, *SNO1*, identified previously in screens of the yeast knockout (YKO) library for mitotic fidelity genes. We found that the causative factor increasing mitotic error rate in the *sno1Δ* mutant is not loss of the Sno1 protein, but rather perturbation to the mRNA of the neighboring convergent gene, *CTF13*, encoding an essential component for forming the yeast kinetochore. This is caused by a combination of the Kanamycin resistance gene and the transcriptional terminator used in the YKO library affecting the *CTF13* mRNA level and quality . We further provide a list of gene pairs potentially subjected to this artifact, which may be useful for accurate phenotypic interpretation of YKO mutants.

## Introduction

Cells must duplicate and equally partition their genomes with high fidelity. Diseases such as cancer are characterized by the disruption of processes involved in maintaining genome stability (Negrini *et al.* 2010). Chromosome instability (CIN), referring to increased rates of chromosome missegregation, is a hallmark of most solid tumors and plays a role in tumor evolution (Bakhoum *et al.* 2018; Potapova *et al.* 2013; Watkins *et al.* 2020). As such, it is of importance to understand the genetic pathways responsible for controlling accurate chromosome segregation.

Using budding yeast as a model eukaryotic cell to study CIN has advantages as this system is easily paired with whole genome screening. Considerable progress has been made toward identifying genes responsible for suppressing CIN using classic screening tools such as the chromosome transmission fidelity (CTF), A-like faker, and loss of heterozygosity assays (Novoa *et al.* 2018; Spencer *et al.* 1990;). At least 692 yeast genes were found to be involved in chromosome segregation in studies using these tools (Stirling *et al.* 2011; Yuen *et al.* 2007). One outstanding challenge is to characterize the roles of unanticipated and uncharacterized hits from these studies and learn how they may act to suppress CIN. The unexpected genes from these screens have the potential to shed light on previously unknown physiological processes affecting genome stability and lead research toward new potential targets or therapies for high CIN diseases.

One such gene that has consistently appeared in budding yeast screens related to altered CIN as well as genome instability is *SNO1* (Novarina *et al.* 2020;Stirling *et al.* 2011; Yuen *et al.* 2007). Sno1 is annotated as a protein of uncharacterized function on the *Saccharomyces* Genome Database (SGD) with a predicted function in the pyridoxine biosynthesis pathway although its glutaminase activity has only been demonstrated *in vitro* (Dong *et al.* 2004; Padilla *et al.* 1998). We set out to determine what role the Sno1 protein might play in determining the fidelity of chromosome segregation. Here, we demonstrate that the protein is dispensable for mitotic function and its presence is not required for high-fidelity chromosome segregation, but rather a previously unexplored artifact introduced by the generation of the yeast knockout (YKO) deletion collection (Giaever *et al.* 2002) causes increased CIN in the *SNO1* deletion background. Our data suggest that this occurs by altering the 3′ untranslated region (UTR) of mRNA from the neighboring convergent gene, *CTF13*, an essential protein involved in kinetochore formation. Of interest to the budding yeast research community, this YKO library artifact originates from the combination of the Kanamycin resistance gene with the associated *TEF1* terminator sequence and has the potential to affect other convergent gene pairs in the genome-wide deletion library.

## Materials and methods

Yeast strain genotypes can be found in Supplementary Table S1. Polymerase chain reaction (PCR) and real-time quantitative polymerase chain reaction (qPCR) primers can be found in Supplementary Tables S2 and S3, respectively. Relevant plasmids are listed in Supplementary Table S4.

### Construction of mutant strains

In all cases, mutant strains were generated fresh into the qCTF background strain (RLY8492). PCR products for homologous recombination transformations were generated in one of two ways depending on availability of preexisting mutants. If a mutant already existed such as a YKO collection mutant, genomic DNA (gDNA) was extracted from relevant YKO collection mutants and PCR was used to amplify the KO Kanamycin resistance (KanMX originating from pFA6-KanMX4) cassette with 400 base pairs up and downstream the open reading frame (ORF) using 5′/3′ UTR check primers designed using Primers-4-Yeast software (Supplementary Table S2; Yofe and Schuldiner 2014). When generating a novel mutant, we amplified the KanMX gene directly from the pFA6a-KanMX6 plasmid (or the pFA6a-KanMX6-T_ADH1_ plasmid when noted) using 40–80 base pairs homology to the genomic region to be targeted. pFA6-KanMX4 and pFA6a-KanMX6 have identical KanMX sequences including the promoter, Kanamycin resistance gene, and the terminator (Wach 1996). Henceforth, we refer to KanMX4/6 interchangeably as “KanMX.” Deletion mutants with *URA3*, NatMX, and HphMX markers were generated using the same method with specific details described in Supplementary Table S1. Transformants were generated using a standard lithium acetate homologous/PEG/single stranded carrier DNA protocol (Gietz and Schiestl 2007).

### Yeast media

All qCTF strains were grown in SD-Leu (Sunrise Science Products, Cat #: 1707-500) liquid media or solid plates prior to performing qCTF assays to maintain selection of the mini-chromosome (MC). During the 24-h nonselective period of the qCTF assay, cells were grown in SD-Complete liquid media (Sunrise Science Products, Cat #: 1701-500). After transformant generation and selection for inserted selectable markers, all other (non-qCTF background) strains were grown in yeast-extract-peptone-dextrose (YPD) unless otherwise noted.

### qCTF flow cytometry assay

The qCTF assay was performed as previously described (Zhu *et al.* 2015) with the following changes: cells were not sonicated and were analyzed on an Attune NxT Acoustic Focusing Cytometer with Autosampler (Thermo, Cat #: A24861). All transformations of flow cytometry and optical density data to CIN rate were performed as per the previously outlined mathematical formula with the assumption of equaling doubling time between MC+ and MC− cells of individual strains. Each boxplot for a qCTF assay is comprised of a range of 3–12 biological replicates for a single mutant. We sometimes include independently isolated transformants for strains (denoted as #1, #2, #3, etc.).

### RNA extractions

A hot acid–phenol chloroform extraction method was used to isolate total RNA. Overnight cultures of yeast were refreshed in 12–50 mL of media to mid-log phase (OD_600_ ∼0.4–0.6). Then the cultures were harvested to pellets in 2 mL screw-cap tubes and frozen in liquid nitrogen. Cell pellets were thawed and resuspended in 300 μL of lysis buffer (8.4 mM EDTA, 60 mM NaOAc pH 5.5) with 20 μL of 20% SDS and heated at 65°C for 1–3 min; 325 μL of 65°C preheated acid phenol (VWR, Cat #: 0981) was added to samples and incubated while shaking for 15 min at 65°C. Samples were cooled for 5 min on ice and pelleted at 14,000 RPM for 10 min in a tabletop centrifuge at 4°C. The aqueous layer was added to 325 μL fresh room temperature acid phenol and vortexed for 30 s to 1 min followed by a second centrifugation under the same conditions. The aqueous layer was added to 325 μL chloroform (Sigma, Cat #: 288306-100ML) in a fresh 2 mL screw-cap tube. Samples were vortexed for 30 s at room temperature and centrifuged at maximum speed in a tabletop centrifuge for 2 min. The top aqueous layer was transferred into an RNAse-free tube and 3.0 M NaOAc pH 5.5 was added to bring the final concentration to 300 mM followed by addition of one volume of isopropanol to precipitate RNA at −80°C overnight. RNA was pelleted at 14,000 RPM in a tabletop centrifuge at 4°C for 30 min, washed in 70% ethanol, dried, and resuspended in 25–50 μL 10 mM Tris pH 8 on the same day the samples were to be used for cDNA generation.

### qPCR

All total RNA samples were treated with RNAse-free DNAse I (NEB, Cat #: M0303S) for 10–15 min at 37°C followed by the addition of 5 mM EDTA and heat inactivation at 65°C for 10 min. cDNA for qPCR was generated using random hexamer primers and the High-Capacity cDNA Reverse Transcription Kit (Applied Biosystems, Cat #: 4368814). cDNA (corresponding to 1 pg to 100 ng of total RNA) and 130 nM each of forward and reverse primers were added to the 2X PerfeCTa^®^ SYBR^®^ Green FastMix^®^, ROX™ master mix (QuantaBio Cat #: 95073) following dispensing into 384-well plates that were subsequently loaded into a BioRad CFX384 Touch Real-Time PCR Detection System. Minus reverse transcriptase (RT) controls were routinely performed and samples were only included in analysis if this control produced at least a five-cycle difference in average Cq value when compared to the samples with RT. qPCR primer sequences as well as validated efficiencies can be found in Supplementary Table S3. Data were analyzed using the comparative C_T_ method as previously described (Schmittgen and Livak 2008).

### 3′ rapid amplification of cDNA ends

3′ rapid amplification of cDNA ends (3′ RACE) was performed as previously described with the following adjustments (Scotto-Lavino *et al.* 2006). Five micrograms of total RNA was used as substrate for cDNA generation using SuperScript III Reverse Transcriptase (Invitrogen, Cat #: 18080044) with the 52 nucleotide “Q_T_” primer (Scotto-Lavino *et al.* 2006). Q5 High-Fidelity Polymerase (NEB, Cat #: M0491S) was used for subsequent rounds of PCR amplification from the cDNA library using an annealing temperature of 64°C and an extension duration of 3 min at 72°C for 30 cycles. A second, nested PCR using the same cycling parameters was performed to decrease the nonspecific amplification from the first round of PCR using a 1:20 diluted template. All PCR reactions were assembled on ice and transferred directly to preheated thermocyclers at denaturing temperatures. 3′RACE primers can be found in Table S2. Following nested PCR, 3′ RACE PCR products were purified and concentrated (NEB, Cat #: T1030S) prior to sequencing. When noted, 3′ RACE products were gel extracted (NEB, Cat #: T1020S) from the highest signal PCR bands prior to sequencing. Primer MRG316 was used for Sanger sequencing all 3′ RACE PCR products (Supplementary Table S2).

### Western blotting

Overnight cultures of cells were refreshed in YPD to log phase for 3.5–5 h at 30°C. Samples were then normalized to OD_600_ of 1.0 and 2.5 mL was pelleted, washed once with chilled H_2_O, and snap frozen in liquid nitrogen. Cell pellets were thawed and resuspended into 120 μL of 1x Bolt™ LDS sample buffer (Invitrogen, Cat #: B0008) supplemented with 40 mM DTT (Thermo Scientific, Cat #: R0861). Samples were boiled for 10 min and then vortexed at top speed with approximately 100 μL of 0.5 mm diameter glass beads (BioSpec Products, Cat #: 11079105) for 1 min and 30 s at 4°C. Lysates were boiled again for 10 min, spun down at maximum speed in a tabletop centrifuge for 1 min, and the supernatant was removed from the debris and glass beads into a fresh tube; 27 μL of this protein suspension was loaded for each sample into a 10-well 4–12% NuPAGE Bis-Tris gel (Invitrogen, Cat #: NW04120BOX) along with 10 μL Precision Plus Protein™ Dual Color Standard (Bio-Rad, Cat #: 1610374). SDS-PAGE gels were run according to manufacturer’s recommendation. The gel was next transferred to a PVDF membrane (Introgen, Cat #: IB24002) using the iBlot™ 2 transfer system. Membranes were blocked in 5% bovine serum albumin (BSA, Sigma, Cat #: A9647) in 1x TBS with 0.1% Tween-20 (1x TBST) for 1 h at room temperature followed by overnight incubation in primary antibody in blocking solution at 4°C. Pgk1 mouse monoclonal antibody (1:3000 dilution) and HA rabbit monoclonal antibody (1:1000 dilution) were purchased from Invitrogen (Cat #: 459250) and Cell Signaling (Cat #: 3724S), respectively. Following eight washes in 1x TBST, the membrane was then incubated in a 1:5000 dilution in 1x TBST of the species appropriate HRP-linked secondary antibody (Cell Signaling, Cat #: 7074S or 7076S) for 1 h at room temperature. After four 1x TBST washes, membranes were incubated in 2 mL of ECL reagent (Bio-Rad, Cat #: 170-5060) for 1–5 min following membrane exposure on a LI-COR Odyssey^®^ Fc Imaging system. Quantification was performed using Image Studio Lite software.

### Spot dilution assay

Log phase cultures of cells were normalized to an OD_600_ of 0.62 and 200 μL of each culture was pelleted, washed once with sterile H_2_O, resuspended in 200 μL sterile H_2_O, and transferred to a flat bottom 96-well plate; 20 μL of the most concentrated sample was then serially diluted into 180 μL of sterile H_2_O in a new well for a total of four 10-fold dilutions; 5 μL of each dilution was then dropped onto YPD or YPD plates supplemented with 200 mg/L Geneticin (G418; Thermo Scientific, Cat #: 11811023) and grown for 48 h at 30°C prior to image collection.

### Statistical analysis

Statistical analysis for the qCTF assay was performed via a one-way ANOVA test on the mean fold-change for samples using the stat_compare_means() function from the ggpubR package (version 0.2.3) with default significance cutoffs (not significant [ns]: *P* > 0.05, *P ≤ 0.05, **P ≤ 0.01, ***P ≤ 0.001, ****P ≤ 0.0001). If the result of the one-way ANOVA test was significant, then a Tukey’s post hoc test was performed to identify which groups significantly differed from the relevant control strain using the tukey_hsd() function from the rstatix package (version 0.6.0) using the same significance cutoffs listed above. All significance cutoffs overlaid onto plots are generated from the Tukey’s test. Statistical analysis for qPCR and Western blot data was performed using the same statistical testing method. Unless otherwise noted, all qPCR experiments were performed using both biological and technical triplicates. Cq values for technical triplicates for each biological replicate were averaged and these Cq averages were used in the subsequent statistical analysis. Statistical analysis was only performed on qPCR data if there was a complete set of at least three biological replicates.

### Code to extract convergent gene pairs from the SGD

A Python script was written to retrieve a list of all ORFs and chromosomal coordinates from the SGD for S288C reference strain genome. In the resulting table, coding strands for each ORF are denoted by a + or − for the Watson or Crick strand, respectively. To identify convergent gene pairs, the script iteratively compares the coding strand of pairs of genes. If both genes were either +/+ or −/−, meaning they are expressed from the same strand, the algorithm skips to compare the next two gene pairs. If the first gene in the pair is + and the second gene pair is −, then they are labelled convergent and this gene pair including its associated information were stored locally to a .txt file.

### Data availability

All yeast strains and plasmids are available upon request. Source code for the convergent gene pair mining from the SGD is available online (https://github.com/mgordo34/Convergent_Gene_Script). Supplementary Table S1 contains the genotypes of budding yeast strains used in this study and the sources of any strains not generated in the present study as well as the primer sets and templates DNA sequences used for homologous recombination transformations and aliases for relevant strains. Supplementary Table S2 contains a list of primer names and sequences used for PCR, 3′ RACE, and DNA sequencing. Supplementary Table S3 contains qPCR primer sequence sets, the ORFs they anneal to, and their experimentally calculated efficiencies. Supplementary Table S4 contains relevant plasmids developed and used during the course of this study. Supplementary Table S5 contains one of the tables generated from the source code for mining convergent gene pairs from the SGD. This table lists nonoverlapping convergent gene pairs, the number of base pairs between each stop codon, the chromosomal coordinates of each gene, and the chromosome they reside on. The source code also produces a table of overlapping convergent gene pairs. Supplemental Material is available at figshare: https://doi.org/10.25387/g3.14195846.

## Results

### The Sno1 protein is dispensable for high-fidelity chromosome segregation

In a previous study, we developed a quantitative tool for measurements of CTF in yeast called the qCTF assay (Zhu *et al.* 2015). This tool improves upon the standard CTF assay (Spencer *et al.* 1990) for measuring the missegregation rate of a mini-chromosome (MC) by enabling high-throughput experimentation to generate single-cell quantitative data via flow cytometry, as opposed to the standard scoring of colored colonies in the sectoring assay. To construct the qCTF yeast background, a short linear MC made from the left arm of chrIII containing the MATα locus as well as a selectable marker was introduced into a MATa haploid yeast strain. The MATα locus on the MC represses the expression of green fluorescent protein (GFP) fused to the MATa-specific gene, *MFA1*, which is highly expressed in the MATa strain. Upon loss of the MC, GFP molecules irreversibly accumulate in the cell, which allows for the detection of the missegregation event using flow cytometry.

As *SNO1* was implicated in previous screens for genes whose deletion affect CIN, we applied the qCTF assay to validate and quantify the effect of a *SNO1* ORF deletion. We engineered fresh qCTF knockout (KO) strains through homologous recombination gene replacement that introduced a Kanamycin resistance (KanMX) gene in place of the target gene using the same PCR-based deletion strategy used to generate the YKO collection (Baudin *et al.* 1993; Wach *et al.* 1994). We chose to add the KanMX KO alleles directly to the qCTF background strain as opposed to engineering the qCTF components into the KO library due to the accumulation of secondary mutations that are known to occur within the KO library (Puddu *et al.* 2019; Teng *et al.* 2013). Because each of our mutants contained the KanMX gene, we additionally generated a control qCTF strain with a KanMX integrated in the *TRP1* locus as a baseline to compare with our mutants. The resulting strains were used to measure a “CIN rate,” which is defined as the frequency of MC loss/cell divisions. Our control strain had a CIN rate of ∼0.00016, equivalent to 1.6 MC missegregations per every ten thousand divisions, which is comparable to our previous data as well as that of the standard CTF assay (Zhu *et al.* 2015). CIN rates are transformed into fold-changes, which are calculated by dividing the CIN rate of a given mutant by the CIN rate of an appropriate qCTF control strain.

**Figure 1:**
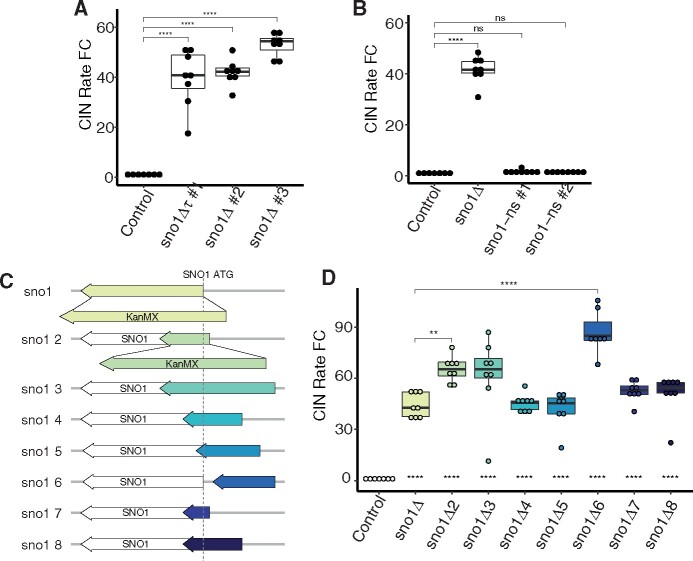
Sno1p is not necessary for high fidelity in chromosome segregation. (A) CIN rate measured using the qCTF assay for three independent isolates of *sno1Δ* mutants compared to a control strain (KanMX inserted into the *TRP1* locus). Boxplot parameters for all qCTF figures are as follows: lower and upper hinges correspond to the first and third quartiles (the 25th and 75th percentiles). The hinge between the upper and lower hinge represents the median of the data. The upper whisker extends from the hinge to the largest value no further than 1.5 * IQR from the hinge (where IQR is the inter-quartile range, or distance between the first and third quartiles). The lower whisker extends from the hinge to the smallest value at most 1.5 * IQR of the hinge. Individual data points are overlaid on the boxplots. P-values are generated from a Tukey’s post hoc test. Default significance cut offs were used as follows: not significant (ns): p > 0.05, *: p ≤ 0.05, **: p ≤ 0.01, ***: p ≤ 0.001, ****: p ≤ 0.0001. (B) CIN rate measurements for two independent isolates of the *sno1-nonsense* (*sno1-ns*) mutant in which Cysteine 84 has been mutated to a stop codon. See Supplementary Figure S1B for Sanger sequencing of gDNA from *sno1-ns* strains to confirm the nonsense point mutation genotype. P-values are generated from a Tukey’s post hoc test. (C) Graphical representation of the *SNO1* locus on chrXIII and corresponding KanMX insertions. The *SNO1* ORF is displayed as a white arrow while KanMX insertions are depicted with colored arrows representing the size of DNA sequence replaced. In each case, the KanMX gene size is consistent, which is represented via blown up KanMX arrows in the *sno1Δ* and *sno1Δ2* cartoons. All other KanMX sequences are represented in shorthand by the colored arrows. All plots are drawn to scale and aligned to the *SNO1* ORF (grey dashed line). (D) CIN rate measurements for the *SNO1* KanMX insertion mutants. P-values indicated as astericks centered at the bottom of each box plot are all pairwise comparisons to the control strain generated from a Tukey’s post hoc test. P-values indicated as astericks and brackets at the top of the graph are pairwise comparisons marking the only KanMX insertion mutants that are significantly different from the *sno1Δ* mutant.

Three independent transformants of a *sno1Δ* mutant showed a ∼40- to 53-fold increase in the CIN rate in the qCTF background (Figure 1A). To validate the functional relevance of the Sno1p in suppressing CIN, we ablated Sno1p function by incorporating a single nonsense point mutation within the *SNO1* ORF at cysteine 84 to prevent full-length protein expression. This mutation was generated through negative selection to generate a “scar-less” genome edit harboring no selectable marker and was sequenced to confirm the presence of the nonsense mutation at the endogenous *SNO1* ORF (Supplementary Figure S1A and S1B; Gray *et al.* 2005). This *sno1-nonsense* point mutant in the qCTF background did not elevate the CIN rate significantly compared to the wild-type control in two independent mutant isolates, suggesting that the high CIN phenotype in the *sno1Δ* mutant was not attributed to the loss of the Sno1p (Figure 1B). We also created a *sno1Δ::URA3* strain, which did not have elevated CIN rates (Supplementary Figure S1A and S1C). Taken together, these data point to a lack of necessity for the Sno1p in maintaining proper chromosome segregation but rather suggest a potential artifact induced by the *sno1Δ::KanMX* mutant of the YKO collection.

When generating the YKO collection, the KanMX gene cassette from the plasmid pFA6a-KanMX encoding aminoglycoside phosphotransferase under the control of the *TEF1* promoter and terminator from *Ashbya gossypii* was used to replace nonessential ORFs (Giaever *et al.* 2002; Wach *et al.* 1994). We therefore hypothesized that the placement of the KanMX gene within the *SNO1* locus, rather than perturbation of the *SNO1* ORF per se, may contribute to the CIN phenotype in this mutant. To test this, we generated a series of KanMX insertions at the *SNO1* locus and measured the CIN rates of these mutants (Figure 1C). These KanMX insertion mutants either deleted portions of the *SNO1* ORF or deleted a portion of the intergenic regions upstream of the *SNO1* ORF. One of these mutants, *sno1Δ6*, had an entirely unperturbed *SNO1* ORF with only 251 base pairs of upstream intergenic sequence containing the *SNO1* promoter replaced with KanMX beginning 51 base pairs upstream of the ORF. All of these mutants significantly increased the CIN rate over 40-fold compared to the control strain, suggesting that the presence of the KanMX gene induces high CIN when inserted at various positions around the *SNO1* locus (Figure 1D). While each of these mutants is significantly different from baseline level of CIN the control strain, only *sno1Δ2* and *sno1Δ6* have CIN rates that are significantly elevated compared with the standard YKO collection *sno1Δ* mutant.

### The expression orientation of KanMX at the *SNO1* locus differentially influences CIN

**Figure 2:**
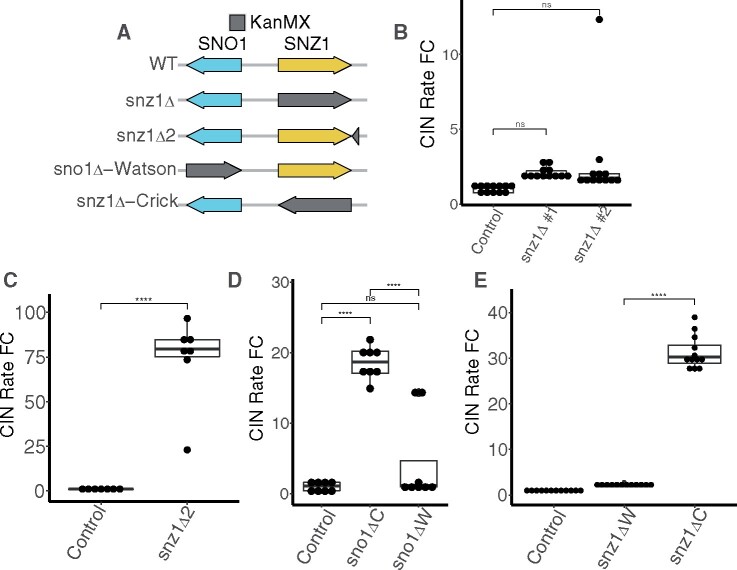
KanMX induces CIN when present at the *SNO1* locus oriented in the same direction as the *SNO1* coding strand. (A) Graphical representation of chrXIII showing the divergent *SNZ1* (yellow) and *SNO1* (blue) gene pair. KanMX deletions are depicted in grey. As noted in Figure 1C, the size of the KanMX is consistent whereas the grey arrow represents the amount of DNA sequence replaced. Right and left pointing arrow orientation represents gene expression from the Watson and Crick strand, respectively. (B) CIN rate measurements for two independent isolates of the *snz1Δ* strain using the qCTF assay. P-values are generated from a Tukey’s post hoc test. (C) CIN rate measurements for the *snz1Δ2* strain using the qCTF assay. P-values are generated from a Tukey’s post hoc test. (D) CIN rate measurements for the a control strain, *sno1Δ-Watson* (*sno1ΔW*) strain, and the conventional *sno1Δ-Crick* (*sno1ΔC*) strain. P-values are generated from a Tukey’s post hoc test. (E) CIN rate measurements for the *snz1Δ-Crick* (*snz1ΔC*) strains compared to conventional *snz1Δ-Watson* (*snz1ΔW*) YKO strain. P-values are generated from a Tukey’s post hoc test.

In an effort to study the *SNO1* locus, we also assayed the CIN rate in a YKO collection mutant deleting the most proximal upstream nonessential gene to *SNO1*, *SNZ1* (Figure 2A). The *SNZ1* and *SNO1* genes are arranged in a divergent orientation and share a common promoter sequence (Padilla *et al.* 1998). The *snz1Δ* CIN rate was not significantly increased compared to the wild-type control (Figure 2B), while a different KanMX insertion downstream of the *SNZ1* ORF in an intergenic region increased CIN more than 70-fold (called *snz1Δ2*, Figure 2, A and C). When an ORF is replaced using the conventional YKO collection method, the KanMX gene is expressed from the same coding strand of DNA as the deleted ORF. For example, the coding strand for *SNO1* is the Crick strand and in the YKO collection *sno1Δ* mutant, the KanMX gene is also expressed from the Crick strand. *SNZ1* is expressed from the opposite, Watson, strand of *SNO1*, which means the KanMX gene in the *snz1Δ* mutant is also expressed from the opposite strand compared to *SNO1*, highlighting a potential difference that may be responsible for the increased CIN observed in the *sno1Δ* mutant.

To determine whether the KanMX expression orientation affects the CIN rate in an *SNO1* deletion mutant, we generated two complementary *sno1Δ* mutants that express KanMX from either the Crick strand (as in the YKO collection) or the Watson strand (Figure 2A, “*sno1Δ-Watson”*). Only the *SNO1* deletion expressing KanMX from the Crick orientation causes a significant increase in the CIN rate compared to the control for this experiment (Figure 2D). Similarly, switching the *snz1Δ* allele such that the KanMX gene is encoded in the Crick orientation raised the CIN rate significantly higher than the *snz1Δ* mutant from the YKO collection (Figure 2E). We further tested the effect of altering the KanMX expression orientation in the *sno1Δ6* mutant, which has an unperturbed *SNO1* ORF coding sequence, and found that the majority of the elevated CIN was significantly reduced. However, the CIN rate was still elevated ∼14- to 19-fold compared to the control levels (Supplementary Figure S2A). The remainder of elevated CIN in the *sno1Δ6-Watson* mutant points to an additional source of CIN independent of KanMX orientation within the *sno1Δ6* background, consistent with the significantly different CIN rate between the *sno1Δ* and *sno1Δ6* reported earlier (Figure 1D).

Finally, the effect of KanMX gene orientation on CIN does not affect all genes that are known CIN mutants, as when we reverse the KanMX gene direction in a different CIN mutant, *csm3Δ*, there was no significant difference in the CIN rate from the conventional KO allele (Supplementary Figure S2B). Together, these findings suggest a localized *cis* effect of the KanMX gene cassette on the CIN rate at the *SNO1* locus with coding expression from the Crick strand, rather than a direct effect due to the absence of the Sno1p. This *cis* effect is present when KanMX is inserted at distances up to 1.3 kilobase pairs upstream of the *SNO1* ORF as in the *snz1Δ2* mutant, which was the farthest distance we tested (Figure 2, A and C).

### *sno1Δ* affects the abundance of mRNA from the downstream gene *CTF13*

**Figure 3:**
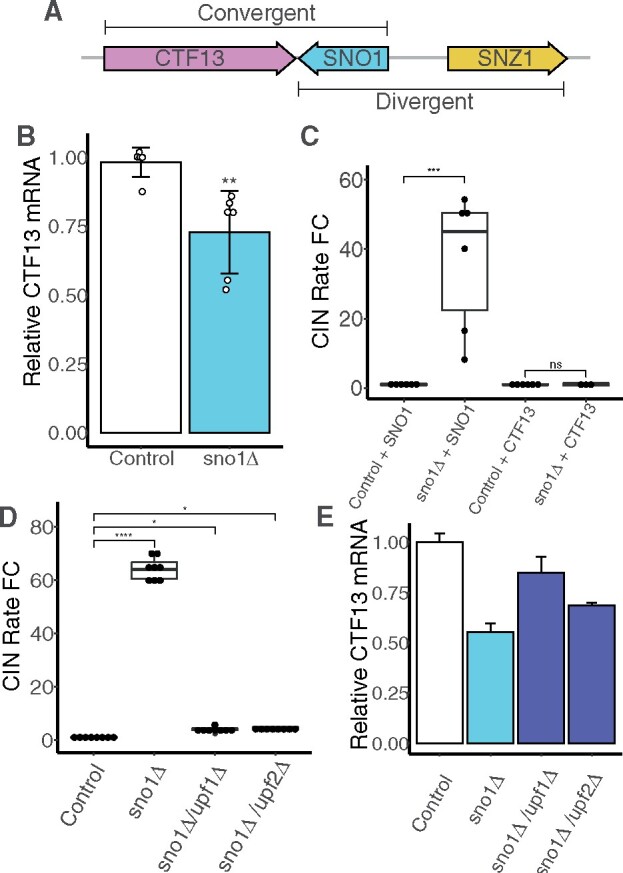
*CTF13* mRNA abundance is reduced in the *sno1Δ* mutant. (A) Graphical representation of the locus on ChrXIII containing *CTF13* (pink), *SNO1* (blue), and *SNZ1* (yellow) drawn to scale. *SNO1* and *CTF13* are convergent gene pairs. *SNO1* and *SNZ1* are divergent gene pairs sharing a common promoter sequence. (B) qPCR analysis of *CTF13* mRNA levels expressed as fold changes performed on control and *sno1Δ* strains using a *CTF13* ORF primer set (set #3, see Supplementary Table S3). Six independent replicates were performed each including technical triplicates. Each values is normalized to the level of *ACT1* mRNA. Technical triplicates are averaged and pooled together and standard deviation is calculated for Cq means of the independent strains. P-values are generated from a Tukey’s post hoc test. (C) CIN rate measurements for control strain and *sno1Δ* strain with either an additional copy of *SNO1* or *CTF13* integrated into an ectopic position on chrIV. P-values are generated from a Tukey’s post hoc test. (D) CIN rate measurements for *sno1Δ* and NMDΔ double mutants (*sno1Δ/upf1Δ* and *sno1Δ/upf2Δ*). P-values are generated from a Tukey’s post hoc test. (E) qPCR analysis of *CTF13* mRNA extracted from strains listed in Figure 3D using a *CTF13* ORF primer set (set #3, see Supplementary Table S3). Only one independent isolate for each strain was used in this experiment and the standard deviation is measured from the technical triplicates. This result was validated using the same cDNA samples with *CTF13* primer set #1 (data not shown).

Given that Sno1p is not responsible for preventing CIN, we next determined whether there may be neighboring elements that are disrupted in the *sno1Δ* mutant and lead to increased CIN. Aside from *SNZ1*, the next closest ORF to *SNO1* is the downstream gene, *CTF13*. *CTF13* is an essential yeast gene encoding a component of the CBF3 inner kinetochore complex that binds to the CDEIII centromere element (Biggins 2013). We noted that *SNO1* is arranged in a “tail-to-tail” (also referred to as convergent) orientation with *CTF13*, such that the 3′ UTRs of both genes are overlapping with only 23 base pairs of intergenic sequence separating the two genes’ stop codons (Figure 3A). Given its close proximity to the *SNO1* gene, *CTF13* mRNA may be altered in the YKO *sno1*Δ mutant. Because *CTF13* is annotated as both a haploinsufficient and an essential gene in the SGD, a reduced abundance of *CTF13* mRNA could explain the heightened CIN in a *sno1*Δ mutant from the YKO collection. To test this, we performed reverse transcription quantitative PCR (qPCR) to estimate the relative abundance *CTF13* mRNA in the *sno1Δ* strain. When compared with the wild-type control, the *sno1Δ* strains showed significant reduction in *CTF13* mRNA levels (Figure 3B). This trend was consistent for a second independent qPCR primer set probing the *CTF13* ORF (Supplementary Figure S3A). Furthermore, we found this trend of reduced *CTF13* mRNA to be consistent in the *sno1Δ6* mutant, which also had increased CIN rates due to the KanMX insertion whereas the *sno1Δ::URA3* mutant had wild-type abundance of *CTF13* mRNA (Supplementary Figure S3B and S3C). These findings suggest that the KanMX sequence, inserted at varying positions in the *SNO1* locus, perturbs the abundance *CTF13* mRNA independent of an unaltered copy of the *SNO1* ORF.

If the CIN phenotype of the *sno1Δ* mutant was due to reduced *CTF13* mRNA, increased dosage of the *CTF13* gene should prevent high CIN phenotype observed in this background. To test this, we examined the effect of adding an extra copy of the *CTF13* gene in the *sno1Δ* background into an intergenic location on chromosome IV (native *CTF13* is located on chromosome XIII). Adding the second copy of the *CTF13* ORF to *sno1Δ* completely suppressed the high CIN back to the wild-type level (Figure 3C). On the other hand, integrating a copy of the *SNO1* ORF into the same location on chromosome IV in the *sno1Δ* background was not sufficient to rescue the high CIN phenotype (Figure 3C). This supports the idea that the causative factor increasing CIN in the *sno1Δ* mutant is reduced *CTF13* mRNA.

### Disrupting the nonsense-mediated decay pathway increases *CTF13* mRNA level and reduces CIN in *sno1Δ* mutants

*CTF13* mRNA is known to be affected by the nonsense-mediated decay (NMD) pathway such that inactivating NMD leads to increased *CTF13* mRNA (Dahlseid *et al.* 1998). This is thought to occur bya mechanism independent of altering the half-life of the *CTF13* mRNA but rather through affecting other transcripts involved in *CTF13* gene expression. Indeed, NMD deletion (*upf1Δ*) led to a fivefold increase in the *CTF13* mRNA when averaged across two independent *CTF13* qPCR primer sets (Supplementary Figure S3D). We therefore reasoned that inactivating the NMD pathway may result in increased abundance of the *CTF13* mRNA to a level sufficient to reduce the CIN phenotype in the *sno1Δ* YKO collection mutant. To this end, we constructed double mutants with *sno1Δ* combined with deletion of either *UPF1* or *UPF2*, genes encoding two of the essential components of the NMD pathway. The resulting double mutants had markedly reduced CIN rates much closer to that of the control strain (Figure 3D). qPCR confirmed that the *CTF13* mRNA abundance was indeed increased in *sno1Δ upf1Δ* and *sno1Δ upf2Δ* double mutants compared to the *sno1Δ* single mutant, although not back to the wild-type levels (Figure 3E).

We also used several KanMX insertion mutants to determine whether inactivated NMD could also suppress the CIN in these backgrounds. In our high-throughput qCTF analysis, *sno1Δ2* and the *sno1Δ6* were found to have significantly increased CIN even compared to the *sno1Δ* mutant (Figure 1D), but *sno1Δ3* does not. This was surprising as *sno1Δ3* deletes a portion of the *SNO1* locus that encompasses both the sno1Δ2 and the sno1Δ6 deleted sequences plus an extra 97 base pair between the two. Our secondary analysis revealed that *sno1Δ3* mutant also had significantly elevated CIN compared to the *sno1Δ* strain (Supplementary Figure S3E). NMD inactivation in either the *sno1Δ2* or the *sno1Δ6* mutant completely suppressed CIN back to the control levels, whereas the *sno1Δ3* CIN rate was unaltered (Supplementary Figure S3E). NMD inactivation led to an increase in *CTF13* mRNA compared to the single *sno1Δ6* mutant (Supplementary Figure S3C), consistent with CIN suppression. This suggests that loss of the sequence uniquely deleted in the *sno1Δ3* mutant has an additional effect on *CTF13* gene expression unrelated to NMD (see Discussion).

### *CTF13* mRNA is abnormally truncated in the *sno1Δ* background

**Figure 4:**
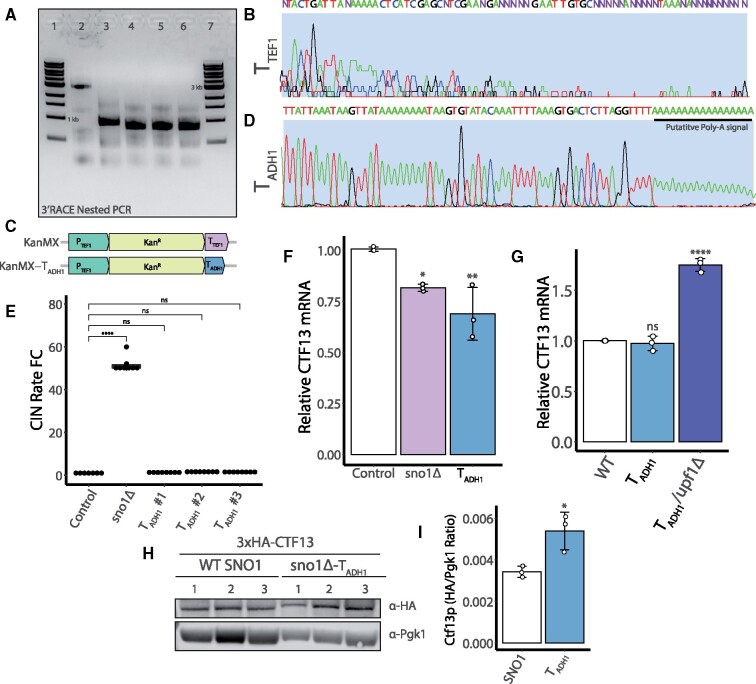
*CTF13* mRNA length and 3′ end composition are altered in the *sno1Δ* mutant. (A) Nested reverse transcriptase (RT)-PCR products from a 3′ RACE assay using a *CTF13* gene specific primer (MRG577) and Q_I_ primer (see Supplementary Table S2) run on a 1% agarose gel stained with ethidium bromide. Lanes 1 + 7: 5 μL 1 kb ladder (NEB # N3232L). Lane 2: Control strain, wild-type *SNO1-CTF13* locus. Lane 3: *sno1Δ::Kan*, Lanes 4-6: 3 independent isolates of *sno1Δ::Kan-T_ADH1_*_._ All strains were engineered into the qCTF background. (B) Sanger sequencing of gel extracted 3′ RACE PCR products for the *sno1Δ* strain trimmed to a 76 base pair window within the *CTF13* 3′ UTR. This includes the final base calls from this sequenced fragment. Purple Ns are reads that could not be accurately assigned using the sangerseqR package in R (Hill *et al.* 2014). Full Sanger sequencing data is available in Figure S5. (C) Graphical representation of the two plasmids used to generate *sno1Δ* mutants. Plasmid maps are displayed as linear fragments zoomed into the KanMX promoter, gene, and terminator segment. pFA6a-KanMX (top) contains the *TEF1* promoter and terminator (P_TEF1_ and T_TEF1_, respectively) sequence from *A. gossypii*. pFA6a-KanMX-T_ADH1_ (bottom, referred to as MGP026 in Supplementary Table S4) was cloned using the pFA6a-KanMX as a backbone and the T_TEF1_ was replaced with a 166 base pair *ADH1* terminator (T_ADH1_) sequence from *S. cerevisiae*. Maps drawn to scale. (D) Sanger sequencing of the 3′ RACE product for the *CTF13* mRNA in the *sno1Δ::Kan-T_ADH1_* strain trimmed to a 78 base pair window within the *CTF13* 3′ UTR. The putative polyA tail signal is shown underlined in black. Full Sanger sequencing results available in Supplementary Figure S6. A black bar is used to indicate the putative polyA reads from this sequencing experiment. (E) qCTF assay CIN rate measurements from three independent isolates of the *sno1Δ::Kan-T_ADH1_* (T_ADH1_ #1-3) strain and *sno1Δ* strain. P-values are generated from a Tukey’s post hoc test. (F) qPCR analysis of *CTF13* mRNA levels performed on control*, sno1Δ*, and *sno1Δ::Kan-T_ADH1_* (T_ADH1_) strains using a *CTF13* ORF primer sets (set #3, see Supplementary Table S3). Three independent replicates were performed with technical triplicates. Each values is normalized to the level of *ACT1* mRNA. Technical triplicates are averaged and pooled together and standard deviation is calculated for means of the independent strains. P-values are generated from a Tukey’s post hoc test. (G) qPCR analysis of *CTF13* mRNA levels expressed as fold changes performed on control and *sno1Δ::Kan-T_ADH1_* (labeled *T_ADH1_* for shorthand) in the presence and absence of functional NMD using a *CTF13* ORF primer set (set #3, see Supplementary Table S3). Three independent replicates were performed each including technical triplicates. Each values is normalized to the level of *ACT1* mRNA. Technical triplicates are averaged and pooled together and standard deviation is calculated for means of the independent strains. Statistical analysis is generated from a Tukey’s post hoc test. (H) Western blot analysis for *3xHA-CTF13* protein levels in a *SNO1* WT background or a *sno1Δ::Kan-T_ADH1_* background. Pgk1 was used as a loading control for quantification in Figure 4I. Exposure times were as follows: 5 min and 10 s for α-HA blot, 1 min and 2 s for α-Pgk1 blot. Uncropped membrane images including the molecular weight are provided in Figure S8E). (I) Quantification of the membrane shown in Figure 4H. Ratios between the HA and Pgkl signal were determined using Image Studio Lite. Statistical analysis was performed using a Tukey’s post hoc test and error bars represent the standard deviation.

Several yeast terminators have been noted to function bidirectionally such that they can be read from either the sense or antisense strand (Heidmann *et al.* 1992; Uwimana *et al.* 2017). The bidirectionality is proposed to have evolved to keep transcriptional noise reduced in a genome that is highly compacted. The *TEF1* terminator from the pFA6a-KanMX cassette used in the YKO library may be read bidirectionally such that the *CTF13* transcript is prematurely terminated in the *sno1Δ* background. This hypothesis predicts two changes to the *CTF13* mRNA in the *sno1Δ* background. First, the transcript would have a different length compared to the wild-type transcript. Second, the composition of the 3′ UTR would be altered. To address both of these predictions, we used 3′ RACE to map the sequence and length of the 3′ end of the *CTF13* transcript in both the wild-type and *sno1Δ* background (Scotto-Lavino *et al.* 2006). *CTF13* is known to have an unusually long 3′ UTR estimated to be greater than 2 kilobases (Dahlseid *et al.* 1998), which we were able to detect using the 3′ RACE method combined with nested PCR (Figure 4A, lane 2). When we assayed the length of the *CTF13* 3′ UTR in the *sno1Δ* mutant, a smaller 3′ UTR was observed as a faster migrating PCR product, supporting the notion that the KanMX gene cassette imparts an ectopic transcriptional termination sequence that truncates the *CTF13* mRNA (Figure 4A, lane 3). Upon sequencing the 3′ UTR of the truncated *CTF13* mRNA transcript in the *sno1Δ* mutant, we found no evidence of a polyA tail but rather we found heterogeneous mRNA ends, evidenced by unclear sequencing reads (Figure 4B, Full Sanger sequencing in S5). 3′ UTR sequence dictates mRNA stability as well as translational efficiency, providing a possible link to explain reduced *CTF13* mRNA and even reduced Ctf13p.

### Addition of homogenous and polyadenylated *CTF13* transcripts suppresses CIN in the *sno1Δ* background

If the *CTF13* mRNA is unstable in the *sno1Δ* background, we reasoned that generating a homogeneous pool of polyadenylated *CTF13* transcripts should overcome the CIN phenotype in this mutant. As such, we replaced the T_TEF1_ from the common plasmid used to generate YKO collection mutants, pFA6a-KanMX, with a budding yeast *ADH1* terminator (T_ADH1_) and generated a novel *sno1Δ* mutant (henceforth called *sno1Δ::Kan-T_ADH1_*, Figure 4C). We chose the T_ADH1_ as it is known to create polyadenylated transcripts as well as function bidirectionally in budding yeast (Heidmann *et al.* 1992; Uwimana *et al.* 2017). Using the same PCR-based 3′ RACE technique, we confirmed that the *CTF13* transcript was indeed truncated in the *sno1Δ::Kan-T_ADH1_* strain (Figure 4A, lanes 4–6) and that it contained a clear polyA signal at the 3′ end (Figure 4D, Full Sanger sequencing in Figure S6).

In this new *sno1Δ::Kan-T_ADH1_* mutant with polyadenylated *CTF13* transcripts, the CIN rate was indistinguishable from the control strain (Figure 4E). This result implies that shortened length of the *CTF13* transcript alone is insufficient to increased CIN. qPCR revealed that the *CTF13* mRNA levels were significantly reduced in the *sno1Δ::Kan-T_ADH1_* strain such that the amount of *CTF13* mRNA was not significantly different between the *sno1Δ* and *sno1Δ::Kan-T_ADH1_* strains (Figure 4F). However, when the *sno1Δ::Kan-T_ADH1_* strain was tested at a later time point, the cells had accumulated near wild-type levels of *CTF13* mRNA (Figure 4G), suggesting that the initial observation of reduced *CTF13* mRNA may be a transient state and the strain eventually accumulate more *CTF13* transcripts. Notably, we never observed *sno1Δ* mutants with wild-type levels of *CTF13* mRNA during the course of this study, further supporting the sustained instability of the *CTF13* transcript in that background. Additionally, disruption of NMD in the *sno1Δ::Kan-T_ADH1_* strain led to an increase in the relative abundance of *CTF13* mRNA (Figure 4G).

Given that the *CTF13* 3′ UTR appears to be efficiently polyadenylated with the bidirectional T_ADH1_ sequence downstream of *CTF13*, we next asked whether a homogenous pool of polyadenylated *CTF13* transcripts within a *sno1Δ6* mutant would be sufficient to overcome the more penetrant CIN increase in this background. To this end, we inserted the 166 base pair T_ADH1_ sequence between *SNO1* and *CTF13* using the “scar-less” method of negative selection (Supplementary Figure S4A, “*CTF13-T_ADH1_*”). Sequencing a PCR product from genomic DNA and 3′ RACE were used confirm the presence of the terminator sequence and that the *CTF13* transcript was both truncated and polyadenylated (Supplementary Figure S4B). The presence of the *CTF13-T_ADH1_* sequence was sufficient to replenish the *CTF13* mRNA back to wild-type levels in the *sno1Δ6* background as well as reduce the CIN rate back to wild-type levels (Supplementary Figure S4C and S4D). This result suggests that the all of the CIN in the *sno1Δ6* mutant can be explained by *CTF13* mRNA defects.

### The combination of the KanMX and *TEF1* terminator affects both the steady-state quantity and quality of *CTF13* mRNA in *sno1Δ* mutants

The above results suggest that the T_TEF1_ sequence is necessary to cause elevated CIN when the KanMX gene is present in the Crick orientation at the *SNO1* locus. To test whether T_TEF1_ sequence is sufficient to induce CIN, we generated *SNO1* deletions using two other markers, pFA6a-NatMX and pFA6a-HphMX, which confer resistance to the drugs nourseothricin and hygromycin, respectively (Supplementary Figure S7A). Importantly, both of these KO cassettes use the same promoter as KanMX and T_TEF1_ immediately following the drug resistance gene as in the pFA6a-KanMX plasmid (Supplementary Figure S7A and S7B).

3′ RACE first confirmed that the *CTF13* mRNA in each of these strains was truncated (Supplementary Figure S7C). We also confirmed that the *CTF13* mRNA was truncated in the *sno1Δ::URA3* strain, indicating that P_URA3_ also functions bidirectionally (Supplementary Figure S7C, lane 4). Surprisingly, neither the *sno1Δ::Nat* mutant nor the *sno1Δ::Hph* mutant had elevated CIN rates as was observed in the *sno1Δ::Kan* mutant (Supplemental Figure S7D), suggesting the T_TEF1_ sequence is insufficient to cause increased CIN. The *sno1Δ::Nat* mutant had wild-type levels of *CTF13* mRNA (Supplementary Figure S7D), whereas the *sno1Δ::Hyg* mutant had reduced abundance of the *CTF13* mRNA similar to what was observed in the *sno1Δ::Kan* mutant (Supplemental Figure S7E). We sequenced 3′ RACE cDNA ends to determine potential qualitative differences in *CTF13* mRNA among these strains. All three biological replicates of the *sno1Δ::Nat CTF13* mRNA had a consistent sequence at the end of the 3′ UTR (Supplemental Figure S7F) corresponding to T_TEF1_ sequence, whereas in the *sno1Δ::Hyg* mutant, two of three replicates matched the same *CTF13* 3′ UTR sequence of the *sno1Δ::Nat* mutant, while one replicate indicated a longer heterogenous *CTF13* mRNA end (Supplementary Figure S7G and S7H). These data suggest that in the *sno1Δ::Kan* (and occasionally in the *sno1Δ::Hyg* strain), the *CTF13* transcript extends into the drug resistance marker ORF. This seems to correlate with transcript instability, but the causal relationship is unclear. This observation highlights the complexity of mRNA perturbation by T_TEF1_ and suggests that some sequence features of KanMX may also be involved.

### Presence of the T_ADH1_ sequence is sufficient to increase Ctf13p levels in a *sno1Δ* mutant

To determine whether Ctf13p level may account for the different CIN rates between the *sno1Δ* YKO collection mutant and the *sno1Δ::Kan-T_ADH1_* mutant, we used negative selection to endogenously tag the *CTF13* ORF with 3x hemagglutinin (3xHA; Supplementary Figure S8A). Importantly, we inserted the 3xHA tag at the N-terminus of *CTF13* to avoid adding any extra DNA sequence that may influence the natural *CTF13* 3′ UTR. Following sequencing-based confirmation of the in-frame *3xHA-CTF13* tag at the endogenous ORF (Supplementary Figure S8A), we further generated *sno1Δ* and *sno1Δ::Kan-T_ADH1_* deletions in the *3xHA-CTF13* background. Of note, although colonies formed following the initial generation of the *3xHA-CTF13/sno1Δ* double mutant, we were unable to isolate single colonies on fresh selective agar plates and as such could not accurately genotype the isolates (Supplementary Figure S8B and S8D).

The *3xHA-CTF13* tag itself seems to cause a very mild growth defect compared to an untagged *CTF13* allele. As such it is possible that the 3xHA tag reduces the efficiency of this essential kinetochore protein and or results in increased Ctf13p degradation, which may explain the pronounced defect in combination with a *sno1Δ* (Supplementary Figure S8C). In characterizing this double mutant, we noticed that releasing selective pressure allowed the *3xHA-CTF13/sno1Δ* strain to grow with only a mild growth defect (Supplementary Figure S8C). It is possible that the *sno1Δ* is lethal in the *3xHA-CTF13* background and, if selection is removed, the cell population adapts, perhaps by shutting down expression of KanMX via mutation or epigenetic regulation. Furthermore, if any aneuploids exist in the original transformed population (*i.e.*, disome XIII that contains the *SNO1-CTF13* locus), these strains can have continued growth even in the presence of G418 selection. Unfortunately, we were unable to confirm the synthetic lethality of the *3xHA-CTF13/sno1Δ* strain using tetrad dissection analysis because the close proximity of *SNO1* and *CTF13* would likely limit crossing over during meiosis.

Although we could not confidently generate a pure *3xHA-CTF13/sno1Δ* double mutant, we were able to generate multiple *3xHA-CTF13/sno1Δ::Kan-T_ADH1_* double mutants, which grew qualitatively similarly to the *3xHA-CTF13* background strain and could grow well in the presence of selective pressure (Supplementary Figure S8C and S8D). Comparing the Ctf13p levels between wild-type and *sno1Δ::Kan-T_ADH1_* log phase cultures, Ctf13p levels were elevated in the *sno1Δ::Kan-T_ADH1_* in three repeated experiments (Figure 4, H and I and Supplementary Figure S8E). Overall, the data presented suggest that the elevated Ctf13p in the *sno1Δ::Kan-T_ADH1_* strain is sufficient to buffer against increased CIN.

### Reduced mRNA within other convergent gene pairs in the YKO collection

**Figure 5:**
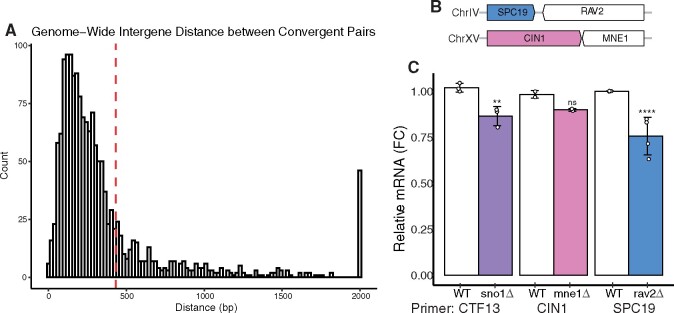
Genome-wide analysis of convergent gene pairs suggests KanMX convergent gene interference can be conserved. (A) Histogram depicting the distribution of intergene distance between all nonoverlapping convergent gene pairs in budding yeast using 100 bins. All distances that are greater than 2000 base pairs are plotted together in a single bin. Red dashed line depicts the median of the dataset at 236 base pairs. (B) Schematic of the *SPC19/RAV2* and the *CIN1/MNE1* gene pairs on their respective chromosomes. Distances are to scale. (C) qPCR analysis of mRNA levels of *CTF13*, *CIN1*, and *SPC19* from *sno1Δ*, *mne1Δ*, and *rav2Δ*, respectively. Three, two, and four independent biological replicates were used for the *CTF13* primer set (set #3), and *MNE1*, *SPC19*, respectively. Each value is normalized to the level of *ACT1* mRNA. Technical triplicates are averaged and pooled together and standard deviation is calculated for means of the independent strains. P-values are generated from a Tukey’s post hoc test.

Although we encountered the KanMX-induced perturbation of mRNA of a neighboring gene in the unique context of the *SNO1* and *CTF13* convergent gene pair, other convergent gene pairs could be affected similarly in the YKO collection. We computationally mined the SGD to extract convergent gene pairs genome wide. This list, which does not include overlapping ORFs, contains 1572 gene pairs encompassing 3144 ORFs and roughly 46.6% of the 6746 ORFs currently annotated in the SGD (Cherry *et al.* 2012; Engel *et al.* 2014; Supplementary Table S5). We further mapped the distribution of the distances between nonoverlapping convergent gene pairs genome-wide and found the median distance to be 236 base pairs (Figure 5A). Although there does not seem to be a minimum distance requirement for the KanMX artifact, there are only 17 convergent gene pairs that have even less intergenic region between stop codons than the *SNO1*-*CTF13* gene pair.

To ask whether other convergent gene pairs may be affected when one gene in the pair is deleted in the YKO, we measured RNA abundance in YKO mutants of two other convergent gene pairs: *RAV2/SPC19* and *MNE1/CIN1* (Figure 5B). The *RAV2* and *SPC19* gene pair is separated by 82 base pairs between their stop codons, while the *MNE1* and *CIN1* gene pair is separated by 30 base pairs. RNA was extracted from *mne1Δ* and *rav2Δ* strains freshly generated using pFA6a-KanMX in the BY4741 background and used primer sets in qPCR experiment for the neighboring convergent gene: *CIN1* and *SPC19*, respectively. Both the *CIN1* and *SPC19* mRNA levels were reduced compared to the wild-type control when the neighboring genes *mne1Δ* or *rav2Δ* were deleted (Figure 5C). This amount of mRNA reduction is similar to what is observed for a freshly generated *sno1Δ* mutant in the same BY4741 background (Figure 5C). Importantly, this result suggests that the *CTF13* mRNA perturbation is conserved among multiple budding yeast strain backgrounds in which *SNO1* is deleted, as all other data were collected using the qCTF background. This neighboring gene reduction was significant in the case of the *rav2Δ* mutant, as is the case in the *sno1Δ* mutant. However, the *CIN1* mRNA reduction in the *mne1Δ* strain was not significant, highlighting the complexity of this T_TEF1_ induced artifact. This result suggests that the *cis* effect observed for the *SNO1-CTF13* gene pair likely occurs to other gene pairs in the YKO collection.

## Discussion

We began our study by characterizing a potential role for *SNO1* in faithful chromosome segregation, as the Sno1p is an uncharacterized protein that consistently appeared in screens using the YKO mutants to assay CIN and genome instability. From our initial experiments, it became clear that Sno1p is not required to suppress CIN, as a nonsense point mutation within the *SNO1* ORF does not elevate the CIN rate. Furthermore, addition of a functional copy of the *SNO1* ORF to a *sno1Δ* background does not decrease the high CIN rate. Evidence points to a specific effect of the KanMX deletion construct used in the YKO collection in elevating the CIN rate of the *sno1Δ* mutant, as three independent selectable markers (*URA3*, HphMX, and NatMX) also disrupting the *SNO1* ORF did not increase CIN. By altering independent portions of the KanMX construct, we were able to pinpoint the increased CIN to a previously unappreciated artifact present within the YKO collection. The transcriptional terminator T_TEF1_ has an ectopic terminator sequence that appears to be read from the opposite strand such that the neighboring gene arranged in a convergent orientation to the deleted gene may have perturbed mRNA levels. The mRNA depletion artifact only occurs when the Kanamycin resistance gene is present upstream of the T_TEF1_, as this phenotype was undetectable or not dominant in the NatMX or HphMX context, respectively.

In the case of *SNO1*, the neighboring convergent gene *CTF13* has truncated and reduced mRNA levels. This seems to underlie the increased CIN in a *sno1Δ* strain, as elevated CIN can be reduced by adding another copy of *CTF13* in a separate genomic locus or by raising the *CTF13* mRNA level via NMD inactivation. Furthermore, this effect appears to be specific to convergent gene pairs, as when we altered the KanMX orientation to be tandem with the *CTF13* gene (as in the *sno1Δ-Watson* or *sno1Δ6-Watson* mutant), CIN was significantly reduced. This observation led us to focus on 3′ UTR composition and determine a difference in the quality of the 3′ ends of the *CTF13* mRNA in the *sno1Δ* mutant, which is truncated and lacks a polyA tail, two factors likely affecting other aspects of the RNA regulation and translation. When *CTF13* mRNA was polyadenylated as in the *sno1Δ::Kan-T_ADH1_* mutant, CIN was suppressed and *CTF13* mRNA eventually reached wild-type levels, which correlated to elevated levels of the Ctf13p.

Given the *CTF13* 3′ UTR is naturally very long and likely encompasses the complete antisense *SNO1* ORF sequence, it is tempting to speculate that the presence of the Sno1p may be irrelevant for chromosome segregation. Instead, the *SNO1* ORF DNA sequence could act as a stabilizer or enhancer for the *CTF13* mRNA. Recent studies have shed light on a novel mode of posttranscriptional control of convergent gene expression through the formation of RNA duplexes that sometimes contain the complete antisense ORF of one mRNA within the 3′ UTR of the convergent gene’s mRNA (Sinturel *et al.* 2015). However, this is not mechanistically relevant for our findings considering that several of the KanMX mutants we screened (*i.e., sno1Δ6, snz1Δ2-Crick*, and *snz1Δ2)* have completely unaltered *SNO1* ORFs but still experience significantly elevated CIN. Furthermore, when a copy of the *CTF13* ORF was added into an endogenous locus in a *sno1Δ* background, CIN was suppressed independent of the presence of a complete *SNO1* ORF. These findings together argue against the necessity for the *SNO1* ORF sequence in maintaining proper chromosome segregation.

Our data confirm previous findings that NMD plays a role in the downregulation of *CTF13* mRNA level, as inactiving components of NMD increased the *CTF13* mRNA level in wild-type, *sno1Δ*, *sno1Δ::Kan-T_ADH1_*, and *sno1Δ6* backgrounds. Given that these mRNAs have different *CTF13* 3′ UTRs as evidenced by 3′ RACE, it is unlikely that each of them would be a direct substrate for NMD. In fact, the natural *CTF13* mRNA was previously proposed to be an indirect substrate of NMD, as the mRNA’s half-life was unchanged in the presence and absence of NMD (Dahlseid *et al.* 1998). It was suggested that transcripts encoding for proteins responsible for *CTF13* transcription are depleted by NMD (Dahlseid *et al.* 1998). NMD inactivation results in differing levels of *CTF13* mRNA in our mutant strains: the *sno1Δupf1Δ* double mutant has sub-wild-type *CTF13* mRNA levels (although increased relative to the *sno1Δ* single mutant) but the *sno1Δ::Kan-T_ADH1_/upf1Δ* double mutant has higher *CTF13* mRNA than the wild-type strain. This difference may reflect that *CTF13* transcription is increased equally in both strains, with extra transcripts retained efficiently in the *sno1Δ::Kan-T_ADH1_* strain due to the stable polyA tail, whereas extra transcripts are still subject to continuous instability in the *sno1Δ* strain.

NMD inactivation did not rescue CIN in *sno1Δ3*, which had an extra 97 base pair deletion compared to *sno1Δ2* and *sno1Δ6* combined. This 97 base pair region has predicted binding motifs for transcription factors encoded by *ASH1*, *AZF1*, *CBF1*, *GCR1*, *PHO4*, and *PHO2* (De Boer and Hughes 2012; Monteiro *et al.* 2020). This sequence also contains the divergent promoter for *SNO1* and *SNZ1*, and some of these transcription factors such as Pho4p are known to regulate the expression of this gene pair (Nishizawa *et al.* 2008). Of particular interest, *GCR1* has a long intron that is known to be regulated by mRNA decay pathways including NMD (Kawashima *et al.* 2014). As such, *GCR1* can encode for multiple RNA and protein isoforms that appear to be regulated by glucose availability (Hossain *et al.* 2016). This finding further highlights the complexity of gene regulation in proximity to *CTF13*.

One outstanding question is the identity of the sequences that may be read from the reverse direction when the KanMX ORF sequence is combined with the *A. gossypii* T_TEF1_ and what molecular players are involved in mRNA perturbation. *A. gossypii* possesses the smallest genome size of any free-living eukaryote at 9.7 megabases (Dietrich *et al.* 2004). The *A. gossypii* genome was found to be highly compacted containing only seven chromosomes with an average distance of 341 base pairs between ORFs. About 95% of *A. gossypii* genes have homologues in *Saccharomyces cerevisiae*. Compared to *S. cerevisiae*, the *A. gossypii* genome has increased gene density and this is believed to be achieved through shortening intergenic regions as opposed to shortening ORFs (Wendland and Walther 2011). The more streamlined and compact *A. gossypii* genome points for an even stronger need to prevent cryptic transcription from the closely spaced neighboring genes and as such it is reasonable to hypothesize that many *A. gossypii* terminators would function bidirectionally.

Although we are currently unsure of the necessary sequence elements within the KanMX cassette that contribute to the mRNA perturbation phenotype, a manual DNA sequence inspection found at least three Nab3p binding sites and one Nrd1p binding site within the T_TEF1_ sequence. Nrd1p and Nab3p function together with the Sen1p helicase to form an transcriptional termination complex that is utilized specifically for short transcripts such as snRNAs, snoRNAs, and cryptic unstable transcripts (Arndt and Reines 2015; Carroll *et al.* 2004; Steinmetz *et al.* 2001). Nrd1p and Nab3p bind the transcript as it emerges from the transcription bubble and act to recruit other factors such as the TRAMP complex and the nuclear exosome. Thus, it is possible that the exosome is targeted to the *CTF13* transcript in the *sno1Δ* background. Indeed, conserved proteins with homology to budding yeast *NRD1* and *NAB3* are both annotated on the *A. gossypii* genome database (Gattiker *et al.* 2007), suggesting this pathway may also exist in *A. gossypii*. However, our results also suggest that the T_TEF1_ sequence was insufficient to cause CIN or reduced *CTF13* mRNA quantity or quality for example when present in the *sno1Δ::Nat* mutant. An additional feature may be that the *CTF13* mRNA in the original *sno1Δ* mutant contains portions of the distal end of the Kanamycin resistance gene ORF in the transcript.

Although the *SNO1* YKO mutant was annotated as having elevated genome instability in each of three independent CIN screening tools (Yuen *et al.* 2007) as well as the qCTF assay as demonstrated in this study, another screen measuring gross chromosomal rearrangements found no difference in a *SNO1-SNZ1* double KO mutant (Kanellis *et al.* 2007). Of note, this study deleted both genes together with a *LEU2* auxotrophic selectable marker and the orientation of the expression of that marker is not reported. Nevertheless, we suspect that the reason this *SNO1* KO mutant from this study does not have increased genomic instability is due to a difference in selectable markers (we do not detect the Nrd1p and Nab3p binding sites in the *LEU2* terminator sequence), a difference in the expression orientation of this marker, or a combination of both scenarios.

The effect that we measured may be extrapolated to other gene pairs arranged in convergent orientations, as the two other example convergent gene pairs we tested had reduced mRNA levels of one gene when the other in the pair was deleted. There is at least one similar example in the literature with the convergent gene pair *VPS36* and *CDC73* on chrXII whose stop codons are separated by 203 base pairs of intergenic sequence. Deletion of all or portions of *CDC73* with KanMX resulted in a subsequent decrease of *VPS36* mRNA levels detected by qPCR (Rodrigues and Lydall 2018). This finding and our data point to an artifact that exists within the YKO collection effecting the interpretation of phenotypes of closely spaced convergent gene pairs. We have compiled a resource of all the genes arranged in a similar convergent/tail-to-tail orientation as a guide for future studies (Supplementary Table S5). Researchers studying YKO mutants for genes in this list should validate phenotypic observations through alternative methods of gene perturbation. To this end, the plasmid generated in this study (pFA6a-KanMX-T_ADH1_) is particularly useful for the community as it contains the universal primer sites, F1 and R1, present on commonly used pFA6a based plasmids that can be used for PCR-based gene replacement.

Of further interest to the yeast research community, our finding raises the possibility of the construction of new partially defective budding yeast mutants in which the KanMX construct is inserted directly after the stop codon in a convergent orientation with specific genes of interest. The concept for these mutants is similar to the Decreased Abundance by mRNA Perturbation (DAmP) mutants (Breslow *et al.* 2008; Schuldiner *et al.* 2005), which place the KanMX construct in a tandem “tail to head” orientation with the gene of interest. In the case of the DAmP mutants, the mRNA perturbation is assumed to be due to the extension of the native 3′ UTR, which is known to perturb mRNA stability (Muhlrad and Parker 1999). The hypothetical convergent KanMX mRNA perturbation would result in an ectopic termination sequence coupled with 3′ UTR heterogeneity based off our current findings.

Finally, other researchers have suggested a concept known as the “neighboring gene effect” within the YKO collection and have created systematic ways to identify genes that may have incorrect annotations due to this problem (Ben-Shitrit *et al.* 2012). However, this resource does not mention a specific issue introduced by the combination of KanMX with the *TEF1* terminator sequence, which can affect the mRNA of neighboring genes within convergent pairs and thus likely contributes to this neighboring gene effect. Without a full understanding of the underlying mechanisms, our findings offer a cautionary tale to the yeast research community.
